# Corrigendum

**DOI:** 10.1002/jcla.24042

**Published:** 2021-10-27

In Volume 33, Issue 1 (2019), Figure 1 was incorrect in the article titled “Tumor suppression effect of targeting periostin with siRNA in a nude mouse model of human laryngeal squamous cell carcinoma” by Dong Ye, Chongchang Zhou, Sijia Wang, Hongxia Deng, and Zhisen Shen.

Incorrect Figure 1

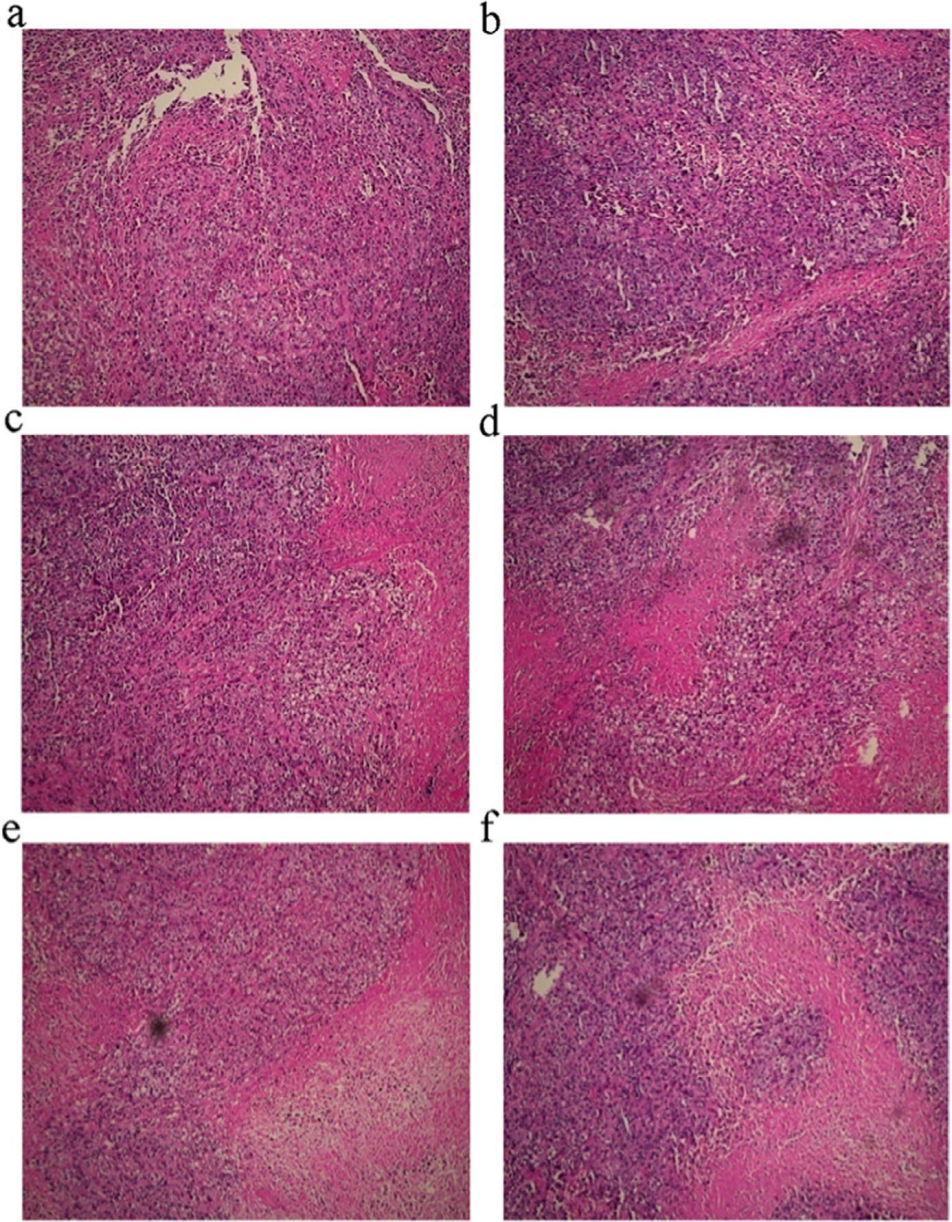

Correct version Figure 1

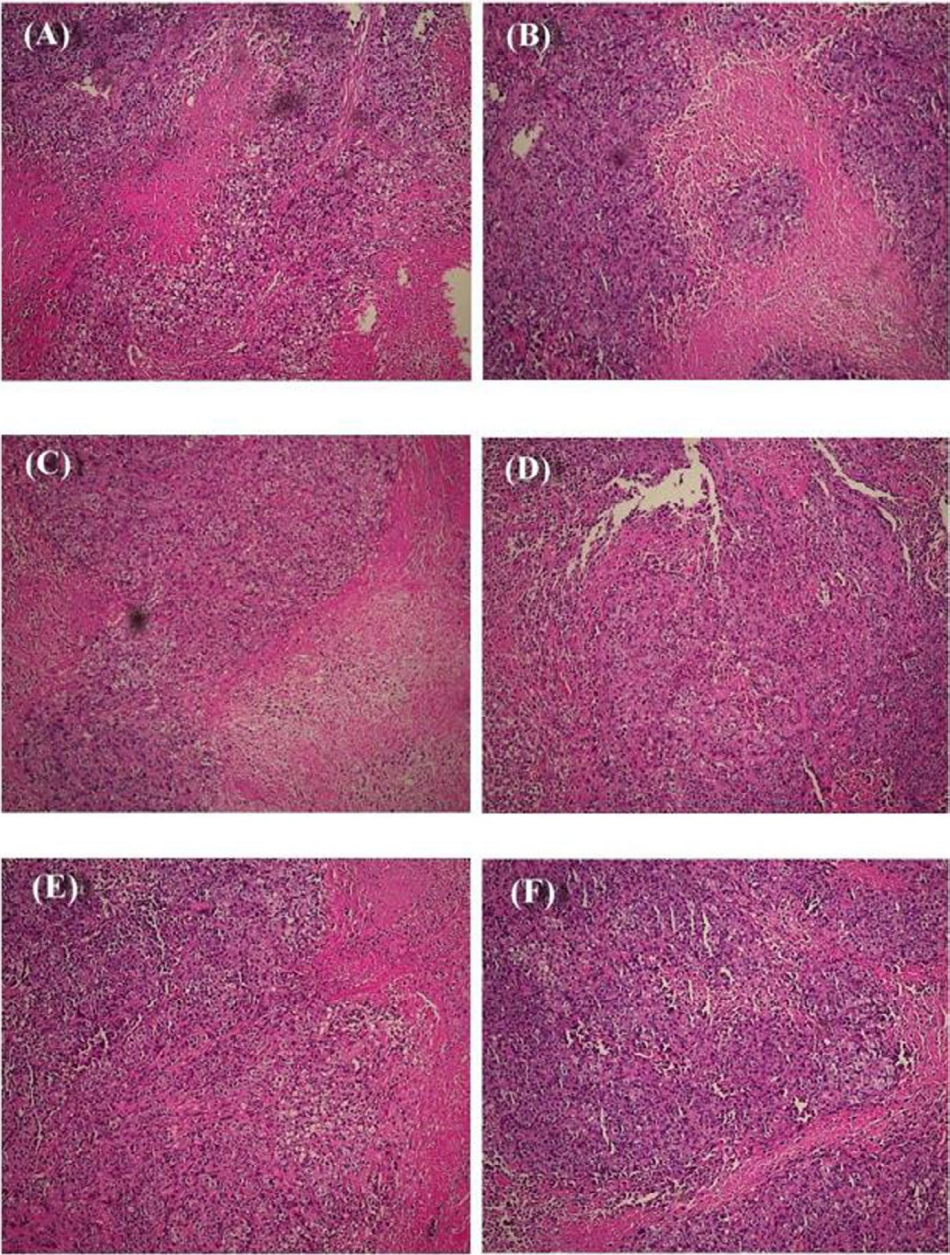

The author regrets this error.

